# Supplementary material for: Predicting visual function by interpreting a neuronal wiring diagram
Source: Nature. 2024 Oct 2;634(8032):113–23. doi: 10.1038/s41586-024-07953-5 (PMC11446822; doi:10.1038/s41586-024-07953-5)
Supplement: Supplementary file 6 — CRF and ERF predictions for individual TmY4 and TmY9 cells. Analogous to Supplementary Data 3, but for TmY target types. Shown are the top four monosynaptic pathways, the strongest pathway passing through each of the top ten intermediary types (ranking from Extended Data Fig. 7), and the trisynaptic pathway Tm1–TmY–Dm3–TmY (see the section entitled Prediction of spatial normalization). [file 41586_2024_7953_MOESM6_ESM.zip › DataS4/TmY9qΓèÑ/720575940622381053.pdf]

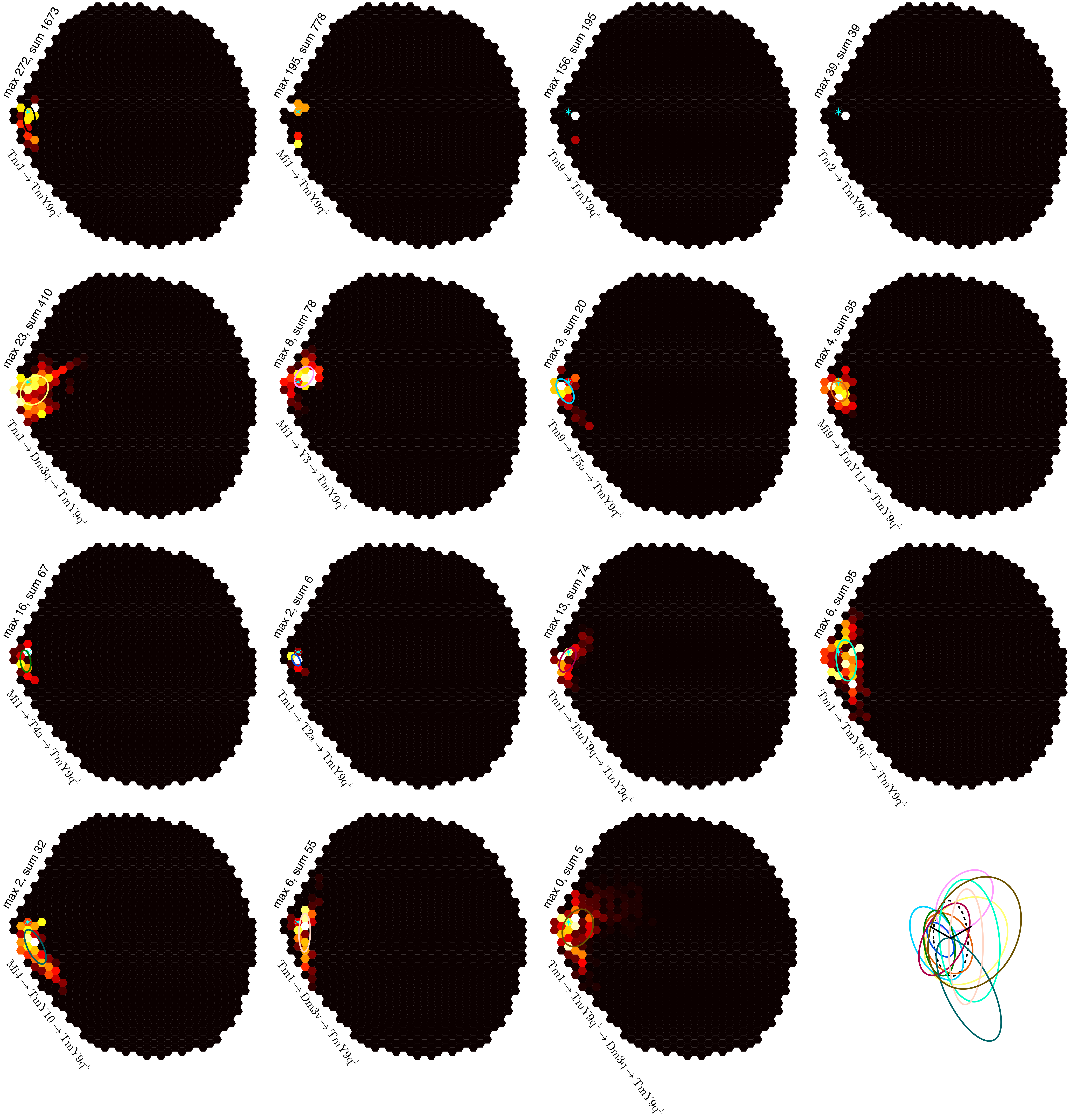
max 272, sum 1673
Tm1 → TmY9q⊥
max 195, sum 778
Mi1 → TmY9q⊥
max 156, sum 195
Tm9 → TmY9q⊥
max 39, sum 39
Tm2 → TmY9q⊥
max 23, sum 410
Tm1 → Dm3q → TmY9q⊥
max 8, sum 78
Mi1 → Y3 → TmY9q⊥
max 3, sum 20
Tm9 → T5a → TmY9q⊥
max 4, sum 35
Mi9 → TmY11 → TmY9q⊥
max 16, sum 67
Mi1 → T4a → TmY9q⊥
max 2, sum 6
Tm1 → T2a → TmY9q⊥
max 13, sum 74
Tm1 → TmY9q → TmY9q⊥
max 6, sum 95
Tm1 → TmY9q⊥ → TmY9q⊥
max 2, sum 32
Mi4 → TmY10 → TmY9q⊥
max 6, sum 55
Tm1 → Dm3v → TmY9q⊥
max 0, sum 5
Tm1 → TmY9q⊥ → Dm3q → TmY9q⊥
